# Suicidality and homelessness: prevalence and associated factors of suicidal behaviour among homeless young adults in Southern Ethiopia

**DOI:** 10.1186/s40359-023-01162-x

**Published:** 2023-04-18

**Authors:** Kalkidan Yohannes, Melkamu Gezahegn, Mekonnen Birhanie, Yilkal Simachew, Awoke Moges, Getinet Ayano, Kusse Koirita Toitole, Hirbaye Mokona, Lulu Abebe

**Affiliations:** 1 Department of Psychiatry, College of Medicine and Health Sciences, Dilla University, Dilla, Ethiopia; 2 SWEDESD, Department of Women’s and Children’s Health, Uppsala University, Uppsala, Sweden; 3 Department of Sociology, Institute of Behavioural science, Dilla University, Dilla, Ethiopia; 4 School of Public Health, College of Medicine and Health Science, Dilla University, Dilla, Ethiopia; 5 School of Public Health, College of Medicine and Health Science, Hawassa University, Hawassa, Ethiopia; 6 Addis Ababa University, Addis Ababa, Ethiopia; 7 Research and Training Department, Amanuel Mental Specialized Hospital, Addis Ababa, Ethiopia; 8 School of Population Health, Curtin University, Perth, WA Australia; 9 Public Health Expert, Doctors with Africa CUAMM, Juba, South Sudan; 10 Department of Psychiatry, College of Medicine and Health Sciences, Addis Ababa University, Addis Ababa, Ethiopia

**Keywords:** Suicidal behaviour, Prevalence, Ethiopia, Rooflessness, Homeless youth, Homeless young adults, Suicidal ideation and attempts, Street homelessness, Low and middle-income countries, Psychiatric emergency

## Abstract

**Background:**

Research indicates that homelessness is associated with an increased risk of suicide. While street homelessness is a global problem, it is a disproportionately serious concern in low- and middle-income countries such as Ethiopia. Despite their high risk of suicidal thoughts and attempts, there has been limited research on this subject among homeless young people in Ethiopia. Therefore, we assessed the prevalence and factors contributing to suicidal behaviour among homeless young people in the southern region of this country.

**Methods:**

We conducted a community-based cross-sectional study from 15 June to 15 August 2020 involving 798 homeless young adults in four southern Ethiopian towns and cities. The Suicide Behaviour Questionnaire-Revised (SBQ-R) was used to assess suicidal behaviour. Data were coded and entered into Epi-Data version 7 and analysed using SPSS version 20. We conducted a multivariable logistic regression analysis to identify factors associated with suicidal behaviour. Variables with a p-value of < 0.05 were considered statistically significant. An adjusted odds ratio with a 95% confidence interval was determined to indicate the association’s strength.

**Results:**

The overall prevalence of suicidal behaviour among young homeless individuals was 38.2% (95% CI: 34.8%, 41.5%). The lifetime prevalence of suicidal ideation, planning and attempt was 10.7% (95% CI: 8.6–12.9%), 5.1% (95% CI: 3.6–6.6%) and 3% (95% CI: 1.9–4.3%), respectively. A longer duration of homelessness (1–2 years) (AOR = 2.244, 95% CI: 1.447–3.481), stressful life events (AOR = 1.655, 95% CI: 1.132–2.418) and the stigma associated with homelessness (AOR = 1.629, 95% CI: 1.149–1.505) were significantly associated with suicidal behaviour.

**Conclusion:**

The results of our study indicate that suicide is a serious public health problem among homeless young people in southern Ethiopia. We have found associations between suicidal behaviour and stressful events, homelessness lasting for one to two years and stigma. Our study suggests that policymakers and programme planners need to develop a strategy for preventing, detecting and managing suicidal behaviour among street-dwelling homeless young adults, a vulnerable and understudied population. A community-based suicide prevention campaign is also essential for street-dwelling homeless young people in Ethiopia.

## Background

Suicide is one of the world’s most serious public health concerns [1]. It refers to the act of deliberately killing oneself [2]. An individual may engage in suicidal behaviour in the form of suicidal ideation (thinking about ending one’s life) [3], a suicidal attempt (non-fatal suicide attempt), or suicide (ending one’s life) [4].

Worldwide, suicide rates are rising [5, 6] which is an alarming global public health issue [1, 2]. According to the World Health Organisation (WHO), close to 800,000 people die from suicide yearly [6]. Based on data from 2019, it was the fourth leading cause of death among people aged 15 to 29. Over half (58%) of all suicides in the world are for people aged under 50 [2]. In addition, most suicide deaths occur in low- and middle-income countries (79%), where most of the world’s population resides [7].

A prior suicide attempt is one of the most significant risk factors for suicide, although there are other factors as well [5, 8, 9]. Nearly one in five young people in low- and middle-income countries have reported suicidal thoughts, making a suicide plan, or attempting suicide within the past year (16.9%, 17.0%, and 17.0%, respectively) [6, 10]. There were several attempts at suicide before each suicide occurred [5]. In addition, for each suicide, there are more than 20 suicide attempts [5].

Youth and young adults are at risk of homelessness at any point in their lives [11], and suicide is one of the leading causes of death among the homeless population [12, 13]. The focus of our research was on street homeless or rough sleepers. Rough sleeping involves sleeping in a public place, such as a street, under a bridge, or in a public place [14]. It can be temporary, seasonal, short-term, or long-term [14]. The UN-Habitat organisation has reported an alarming increase in homelessness over the past decade, with approximately 15 million people being forced out of their homes yearly [15]. It is the most widespread problem among young people [16].

### Suicidal behaviour among homeless

There is a high rate of suicide attempts among youth experiencing homelessness [17–19]. The prevalence of suicidal behaviour among homeless young people varies, as the rate identified depends on the research method, the tools used, and the sample size. Based on a systematic review and meta-analysis, the present and lifetime prevalence of suicidal ideation among homeless individuals is 17.83% and 41.6%, respectively [12]. Suicide is the leading cause of death among homeless youth [13, 20]. Several studies have indicated that 20–68% of homeless youth have attempted suicide at some point [17, 18, 21, 22].

In a follow-up study conducted among 208 homeless youth in New York City and Toronto, the authors found that 46% of participants reported having made at least one suicide attempt in their home or street environment, and 78% reported having attempted suicide more than once [17].

Suicidal thoughts and attempts were measured in a cross-sectional study conducted in the USA among 524 young people experiencing homelessness. The research used items adapted from the Columbia-Suicide Severity Rating Scale (C-SSRS) [23] and the Suicidal Behaviours Questionnaire-Revised (SBQ-R). The survey found that 34% of respondents had attempted suicide at least once in their lifetime [24].

According to another study conducted among 32,010 homeless individuals in Philadelphia, PA, USA, using BASIS-24 (Behavior and Symptom Identification Scale), 24.1% (n = 32) reported suicidal thoughts [25]. A similar study was conducted in the United States in which 1,992 unemployed young adults were asked: “In your entire life, have you attempted suicide?” Twenty-one per cent of respondents reported having attempted suicide in the past year [26].

Another cross-sectional study was conducted among 330 homeless adults in Toronto, Ontario. According to the authors, 61% of the study sample reported having had suicidal thoughts and 34% had attempted suicide [27].

On the other hand, a prospective cohort study of 660 street youth aged 14 to 26 conducted in Vancouver, Canada, found that the prevalence was lowest among street youth. Thirty-five individuals (5.3%) reported having attempted suicide [28].

A cross-sectional study was conducted in Addis Ababa, Ethiopia, to determine the prevalence of mental disorders and unmet needs among homeless individuals living on the streets. According to the study’s authors, 14.8% of homeless adults had attempted suicide the previous month [29].

### Factors associated with suicidal behaviour

Several sociodemographic factors play an essential role in predicting suicide [30], including age [30, 31], gender [30, 32], marital status, family income, level of education, current place of residence, and family status [33]. Researchers have found that several clinical factors can predict suicidal behaviour, including recent physical [34] or mental health conditions [25, 31, 35], treatment-resistant depression [36], alcohol or drug use [25, 31, 35, 37], immune-inflammatory abnormalities [38, 39], a history of mental illness in the family, family history of suicide, and a previous history of suicidal attempts [40].

Homelessness-related factors are significant predictors of suicidal behaviour [37, 41, 42], including the reasons for homelessness, the duration of homelessness, support from an organisation, having a homeless family member, and the number of homeless individuals in a family. According to a large epidemiological survey, suicide attempts in the last year and homelessness are highly correlated [26]. Childhood homelessness and homelessness periods of longer than six months [27] are strong predictors of suicidal behaviour.

The perception of homelessness contributes to low self-esteem, loneliness, feeling trapped, and suicidal thoughts, along with feelings of guilt or self-blame [43]. Evidence indicates that a lack of social support [44] and stressful life events [45], can lead to suicide. The stigma surrounding homelessness [43, 46] is a significant psychosocial predictor of suicide among homeless people [46].

Individuals’ poor social connections with family and friends may contribute to their suicide risk [47, 48]. There is evidence that homeless individuals who attempt suicide have experienced high stress throughout their childhood and adolescence [45, 48].

Suicide has several risk factors, including childhood abuse [28] and street victimisation [21]. Adverse childhood events are associated with suicidal behaviour among youth and young adults, including childhood trauma, types of trauma (verbal, physical, or sexual), and the loss of a parent [28]. Studies on suicide and homelessness among young adults have primarily been conducted in developed countries [49–51].

In Ethiopia, especially in the major cities, the plight of homeless youths and young adults is an undeniable problem, as it is associated with poverty, mental illness [52], urbanisation, disease, and the break-up of families [53–56]. In Ethiopia, there are several government-sponsored mental health scaling-up programmes. Still, it is unlikely that many homeless individuals who suffer from mental disorders have been able to access these services [29].

To our knowledge, there has been no research on suicidal behaviour among street-living homeless young people in Ethiopia. Thus, our study aimed to assess the prevalence and factors associated with suicidal behaviours among street-living youth and young adults in southern Ethiopia. The evidence indicates that members of this highly vulnerable and understudied group exhibit relatively severe mental health outcomes, including suicidal behaviours. As a result, the study will contribute to developing and implementing effective suicide prevention strategies for this group of youth (i.e., street-living homeless youth and young adults).

## Methods and materials

### Study design, setting, and period

We conducted a community-based cross-sectional study. The study was conducted in southern Ethiopia between 15 and 2020 and 15 August 2020 in one city administration (Hawassa city) and three town administrations (Dilla town, Wolaita Sodo town, and Arba Minch town). On 18 June 2020, a distinct region, Sidama, was established from the Southern Nations, Nationalities, and Peoples Region. Hawassa is the capital of the Sidama regional state in Ethiopia’s south region. The city is located 275 km south of the capital of Ethiopia and is the largest in southern Ethiopia. It occupies an area of 157.2 square kilometres, divided into eight sub-cities and 32 Kebeles. Hawassa city comprises Hayek Dare, Menehariya, Tabore, Misrak, Bahile Adarash, Addis Ketema, Hawela Tula, and Mehal Ketema sub-cities.

Dilla is one of the town administrations. A main road crosses the centre of Dilla town, 359 km from Addis Ababa. The town is located in the Gedeo zone of the Southern Nation, Nationalities, and Peoples Region of Ethiopia. It is an active commercial centre and one of the fastest-growing towns in the country. It consists of nine Kebeles (the lowest administrative unit in Ethiopia). No exact information is available concerning the number of homeless young people living on the streets in Dilla town.

Arba Minch is the other town administration study area. It is a town located in the Gamo Gofa Zone of the Southern Nations, Nationalities, and Peoples Region, about 500 km south of Addis Ababa. It comprises four administrative sub-cities, Secha, Sikella, Abaya, and Nechsar, divided into eleven Kebeles.

The fourth study area was Wolaita Sodo town. The town is located 380 km from Addis Ababa. It is the administrative capital of the Wolaita zonal administration in South Ethiopia. The town has three sub-cities, each with 11 lower administrative units.

### Study participants

The study included all street-dwelling, homeless young people aged 15 to 34 who had resided in the study areas (i.e., in the selected city administration and town administrations of southern Ethiopia) for at least six months before the study and were available during the data collection period. We excluded street-living homeless young people who were severely ill and unable to communicate during the study period.

### Sample size determination and sampling procedure

We calculated the sample size in the current study using a single proportion formula under the following assumptions: a 95% confidence interval, a 5% margin of error, and a 50% prevalence of suicidal behaviour among street-dwelling homeless young people in Ethiopia. (i.e., as no published or even unpublished studies have been found in Ethiopia among suicidal behaviour of youth and young people), yielding a minimum sample size of 384.

We then added a 10% non-response rate to the minimum required sample size of 384, yielding a sample size of 423. We then multiplied the sample size by design effect two to increase and decrease the variability introduced by convenience sampling, resulting in a final sample size of 846.

We purposefully selected one city administration (i.e., Hawassa city) and three town administrations (i.e., Dilla town, Wolaita Sodo town, and Arba Minch town) in southern Ethiopia due to the high concentration of street-dwelling homeless youth and young adults in these areas. Then, from each selected city administration and town administration, we selected Kebeles (the smallest administrative unit in Ethiopia) at random. Finally, we used the convenience sampling technique to select participants.

### Data collection and measurement

Nurses with BSc degrees and public health experts conducted face-to-face interviews using structured, pretested, and standard questionnaires. The survey had four sections: the first assessed sociodemographic characteristics (age, gender, marital status, place of birth, educational status, family income, current residence, and family status). A second section assessed clinical factors (recent illness, family history of mental illness, suicide history, depression, and lifetime and current substance use history). In the third section, we examined childhood trauma experiences, forms of trauma (verbal, physical, and sexual), and the death of parents. The last part, the fourth section, examined factors related to homelessness (reasons for homelessness, duration of homelessness, support from an organization, the presence of a family member who is homeless, and the number of homeless); aspects of the psychosocial environment (stigma and stressful life events).

### Suicide behavior questionnaire-revised (SBQ-R)

We measured suicidal behaviour with four items from Suicide Behavior Questioner-R (SBQ-R) [24]. We set a cut-off point of seven, indicating that participants scoring seven or higher exhibited suicidal behaviours. Based on the findings from a validation study of the SBQ-R using clinical and nonclinical samples, the acceptable cut-off scores were 7 for non-suicidal and 8 for clinical samples [24]. Suicidal behaviour was examined in each item (e.g., ideation, planning, threats, and attempts).

In the first question, the respondents were asked if they had ever considered or attempted suicide. Second, the respondents were asked how often, over the previous 12 months, they had thought about suicide (i.e., how often have you thought about killing yourself recently?). A third question inquired about suicide threats (i.e., have you ever told someone you intended to commit suicide or that you might do so?).

In the final question, the participants were asked whether they were likely to engage in suicidal behaviour in the future (how likely is it that one day you will attempt suicide?) [24]. With a cut-off > = 7 in the non-psychiatric general population, the tool has a sensitivity of 93% and a specificity of 95% [24].

### The alcohol, smoking, and substance involvement screening test (ASSIST)

The WHO Alcohol, Smoking and Substance Involvement Screening Test (ASSIST) tool, an eight-item questionnaire developed to assess substance use, was used to measure the presence of substance use [57]. The purpose of ASSIST is to detect psychoactive substance use and related problems among primary care patients and screen adults for problems or risky substance use [58]. The ASSIST can detect various problems associated with substance abuse, including acute intoxication, regular use, dependence, and injecting behaviour.

Each question on the ASSIST has a set of responses, and each response has a numerical score. The Specific Substance Involvement score is calculated by adding the responses to Questions 2–7 for each of the available substances: tobacco, alcohol, khat (amphetamine-type stimulants), and cannabis (marihuana, hashish, ganja). The ASSIST-specific substance involvement scores of ≥ 10 for alcohol and ≥ 4 for any substance indicate problematic substance use [57].

Depression was measured using Patient Health Questionnaire-9 (PHQ-9), a 9-item depression screening and diagnostic questionnaire for MDD based on DSM-IV criteria with a sensitivity of 86% and specificity of 67%. Based on a study conducted in Ethiopia, the PHQ-9 is a reliable and valid instrument for screening MDD in adults [59].

### Data quality management

We trained the data collectors and supervisors on using the data collection tool, procedures, and ethical considerations. The supervisors and principal investigator supervised the data collection process daily, and questionnaire answers were checked for completeness and coding.

In addition, we developed and modified a semi-structured questionnaire to ensure the quality of data collection. We translated a validated English questionnaire into Amharic and then back to English. To protect the privacy of personal information, we have made sure that it is not disclosed to unapproved parties. We used locked cabinets in locked offices for paper-based data and digital data, and we used password-protected computers. The questionnaire was pretested on 5% of the sample to verify its validity. Despite this, we did not modify the outcome measurement tool.

### Data processing and analysis

The data were checked for completeness, coded, entered into Epi-INFO version 7, and exported to SPSS version 20 for analysis. We summarized the data using means, frequencies, and percentages and presented them in figures, tables, and text. Then, we did a bivariate analysis to describe the associations of each independent variable with suicidal behaviour. Variables with a p-value less than 0.2 were considered for the multivariable logistic regression to control the effects of confounding variables. We ran the Hosmer-Lemeshow goodness of fit test for the model, which yielded a p-value of 0.65, indicating a satisfactory fit. Finally, variables with P-values less than 0.05 on a multivariable logistic regression analysis were considered statistically significant and identified using odds ratios (OR) with 95% confidence intervals.

## Results

### Sociodemographic and economic characteristics

The study included 798 participants, with a response rate of 94.32%, with 620 (77.7%) men participating. Three hundred ninety-four participants were under 20 (49.4%), and 650 (81.5%) were single. Most participants (68.3%) attended only a primary educational level, while 13.5% had a secondary level of education. Nevertheless, 18.1% of respondents had no formal education. 80 homeless youth and young adults (10%) did not know their family members. Over a third of the 798 participants had a single parent (35.8%) **(**Table 1**).**

**Table 1:** Socio-demographic characteristics of street homeless young adults in SNNPR, Southern Ethiopia, 2020 (n = 798)

Variables	Categories	Frequency	Percent (%)
Sex	Male	620	77.7
	Female	178	22.3
Age in years	15–19	394	49.4
	20–24	186	23.3
	25–29	98	12.3
	30–34	120	15
Marital status	Married	128	16
	Single	650	81.5
	Divorced/ widowed	20	2.6
Level of education	No formal education	145	18.1
	Primary education	545	68.3
	Secondary education	108	13.5
Family status	Two parents alive	318	39.8
	One parent alive	286	35.8
	Both were died	114	14.3
	He/she don’t know the family	80	10

### Psychosocial and clinical characteristics

A total of 186 respondents (23.3%) reported being recently ill, but the study did not include any severe cases. A total of 259 participants (32.5%) had a family history of substance abuse, and 166 (20.8%) had a family history of suicide. Most participants (90.9%) had no family history of mental illness. One per cent of participants reported having a heart condition, and 107 (13.4%) reported having communicable diseases. It was found that almost half of 343 (43%) of the participants had been abused as children. Most participants (59.7%) also reported experiencing stressful life events during the past six months. Three hundred eighty-six participants (48.4%) had depression **(**Table 2**).**

**Table 2:** Description of psychosocial and clinical characteristics of street homeless young adults in SNNPR, Southern Ethiopia, 2020 (n = 798)

Variables	Categories	Frequency	Percent (%)
Presence of current medical condition	No	612	76.7
	Yes	186	23.3
Family history of substance use	No	539	67.5
	Yes	259	32.5
Family history of suicide	No	632	79.2
	Yes	166	20.8
Family history of mental illness	No	725	90.9
	Yes	73	9.1
History of childhood abuse	No	455	57
	Yes	343	43.
Type and presence of stressful life event in the past 6 months	Car accidens	33	4.1
	Medical/Surgical condition	227	28.4
	Sexual violence	109	13.7
	Death of family	108	13.5
	No experience of stressful life evets	321	40.2
Depression	No	412	51.6
	Yes	386	48.4

### Homelessness-related characteristics

Several factors contributed to street homelessness. Almost one-third of the participants, 247 (31%), had two or more homeless family members, and 485 (60.8%) had experienced the stigma associated with homelessness. Seven hundred sixty-nine participants (96.4%) reported a lack of support from government or non-government organizations. Economic problems accounted for 627 (78.5%), conflict within the family 102 (12.8%), death of a family 42 (5.3%), and others 27 (3.4%). There were 467 cases of those living on the streets for less than six months and 141 cases of those living on the streets for more than two years **(**Table 3**).**

**Table 3:** Description of homelessness-related characteristics of street homeless young adults in SNNPR, Southern Ethiopia, 2020 (n = 798)

Variables	Categories	Frequency	Percent (%)
Homelessness among family members	No family member is homeless	378	47.4
	Presence of one homeless family	173	21.7
	Two or more homeless family members	247	31
Homelessness-related stigma	No	313	39.2
	Yes	485	60.8
Governmental and non-governmental support	No	769	96.4
	Yes	29	3.6
Homelessness duration	< 6 months	467	58.5
	6–12 months	82	10.3
	1-2 years	108	13.5
	> 2 years	141	17.7

### Participants’ current substance use characteristics

Nearly a third (26.3%) of the participants had used tobacco regularly over the preceding three months. A total of 174 street homeless youth and young adults (21.8%) drank alcohol daily at the time of the survey. A total of 206 homeless individuals (25.8%) chewed khat every day for the past three months. One hundred eight (13.5%) participants used inhalants such as spray paints, glue, and benzene.

### Prevalence of suicide behaviours

**Fig. 1:**
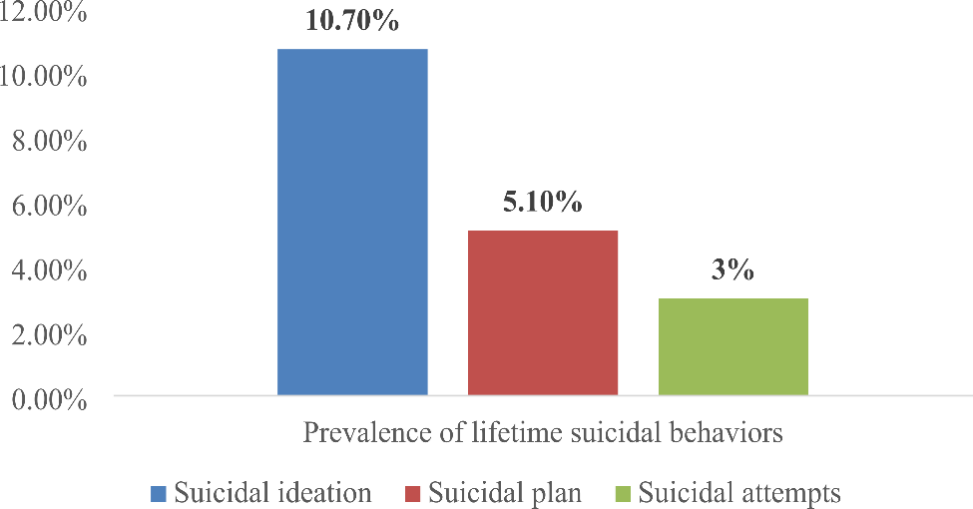
Prevalence of lifetime Suicidal Behavior among street-dwelling homeless young people living in Southern Ethiopia, 2020

**Fig. 2:**
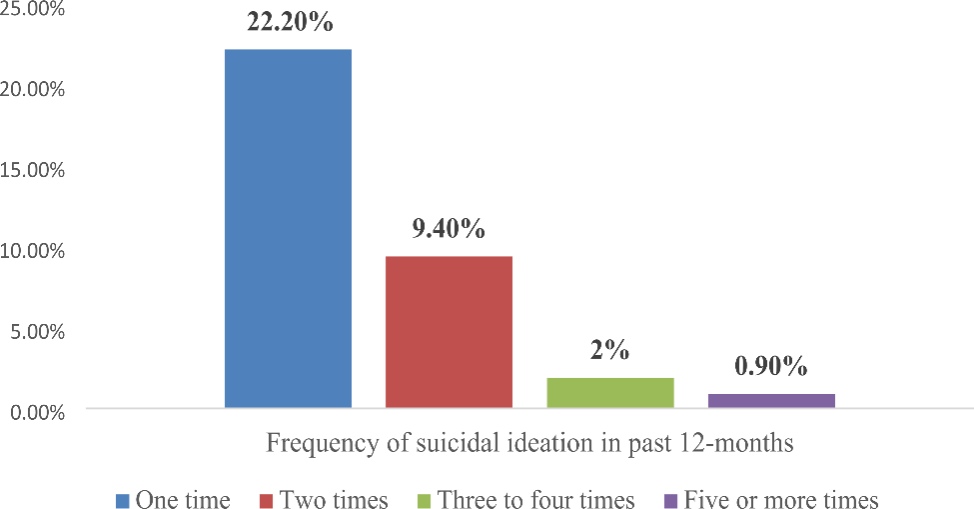
The frequency of suicidal ideation in the past 12-months among street-dwelling homeless young people living in Southern Ethiopia, 2020

In the current study, the prevalence of suicidal behaviour among street-living homeless young people was 38.2% (95% CI: 34.8%, 41.5%). The lifetime prevalence of suicidal ideation, plan and attempt was 10.7% (95% CI: 8.6–12.9%), 5.1% (95% CI: 3.6–6.6%), and 3% (95% CI: 1.9–4.3%), respectively (Fig. 1**).** Moreover, regarding the frequency of suicidal ideation over the past 12 months, 22.2% of study participants reported having experienced it once, 9.4% reported having experienced it twice, 1.9% reported having experienced it three to four times, and 0.9% reported having experienced it five times or more (Fig. 2**).**

### Factors associated with suicidal behaviour

During the bivariate logistic regression analysis, variables such as age, current medical condition, duration of homelessness, stressful life events, homelessness-related stigma, history of childhood abuse, and family history of mental illness were associated with suicidal behaviour (had a p-value less than 0.2). They were entered into a multivariate logistic regression model for further analysis.

In the multivariable logistic regression analysis, the duration of homelessness, stressful life events, and homelessness-related stigma were statistically significant with suicidal behaviour among homeless young people. However, there was no statistically significant difference between street-dwelling homeless youth who engaged in suicidal behaviour and those who did not believe in terms of age, physical condition, history of childhood abuse, or family history of mental illness.

Homeless youth and young adults with a long duration of homelessness (i.e., 1–2 years) were at higher risk for suicidal behaviour as compared to street-dwelling homeless youth with a short period of homelessness (less the six months) (AOR = 2.244, 95% CI: 1.447, 3.481).

Suicidal behaviour was 1.165 times more likely among street-dwelling homeless youth who had experienced stressful life events than those who had not (AOR = 1.655, 95% CI: 1.132–2.418). The street-dwelling homeless youth who had experienced homelessness-related stigma were 1.629 times at risk for suicidal behaviour as compared to those who had not experienced stigma (95% CI: 1.149–2.309) **(**Table 4**).**

**Table 4:** Bivariate and multivariate analysis of factors associated with suicidal behaviour among street homeless young adults in SNNPR, Southern Ethiopia, 2020 (n = 798)

Variables	Categories	Suicidal Behaviour		COR (95% CI)	P value	AOR (95% CI)
		**No**	**Yes**			
Age of participants	15–19 year	258 (65.5%)	136 (34.5%)	0.713 (0.470,1.082)	P = 0.112	0.766 (0.648,1.802)
	20–24 year	109 (58.6%)	77 (41.4%)	0.956 (0.6, 1.521)	P = 0.849	1.008 (0.604, 1.684)
	25–29 year	57 (88.2%)	41 (41.8%)	0.973 (0.567, 1.671)	P = 0.921	1.192 (0.656,2.165)
	30–34 year	69 (57.5%)	51 (42.5%)	1		1
Homelessness related stigma	No	173	140	1		1
	Yes	320	165	0.637 (0.476, 0.853)-	P = 0.002	**1.629 (1.149,1.505)** **P Value = 0.006**
Duration of homelessness	< 6 months	294 (63%)	173 (37%)	1		1
	6–12 months	49 (59.8%)	33 (40.2%)	1.145 (0.708, 1.849)	P = 0.581	1.081 (0.648,1.802)
	1–2 year	47 (43.5%)	61 (56.5%)	2.206 (1.443, 3.371)	P < 0.001	**2.244 (1.447, 3.481)** **P Value = 0.000**
	Above 2 year	103 (73%)	38 (27%)	0.627 (0.413, 0.951) P = 0.028		0.629 (0.409, 0.969) P Value = 0.035
Current Medical illness	No	367 (60%)	245 (40%)	1		1
	Yes	126 (67.7%)	60 (32.3%)	0.713 (0.504, 1.010)	P = 0.057	0.663 (0.438, 1,005)
History of childhood abuse	No	271 (59.6%)	184 (40.4%)	1		1
	Yes	222 (64.7%)	121 (45.3%)	0.803 (0.601, 1.073)	P = 0.138	1.051 (0.734, 1.505)
Family history of mental illness	No	456 (62.9%)	269 (37.1%)	1		1
	Yes	37 (50.7%)	36 (49.3%)	1.649 (1.018, 2.673)	P = 0.042	1.625 (0.975, 2.709)
Stressful Life Events in the past 6 months (SLE)	Road Traffic Accident (RTA)	15 (45.5%)	18 (54.5%)	2.092 (1.016, 4.307)	P = 0.045	1.997 (0.922, 4.322)
	Medical/surgical condition	122 (53.7%)	105 (46.3%)	1.501 (1.062, 2.121)	P = 0.022	**1.655 (1.132, 2.418)** **P Value = 0.009**
	Sexual/other type of violence	77 (70.6%)	32 (29.4%)	0.725 (0.453, 1.160)	P = 0.180	1.243 (0.697, 2.215)
	Death of family	75 (69.4%)	33 (30.6%)	0.767 (0.480, 1.225)	P = 0.267	0.873 (0.513, 1.485)
	No experience of SLE	204 (63.6%)	117 (36.4%)	1		1

Key: * = p-value less than 0.05; COR crude odds ratio, AOR adjusted odds ratio, CI confidence interval

## Discussion

### The prevalence of suicidal behaviour

In this cross-sectional study of street homeless young people, we found high prevalence rates of suicidal behaviour. According to the survey, 38.2 per cent of homeless youth and young adults have reported suicidal behaviour (95% CI = 34.8-41.5%). Suicidal behaviour was associated with stressful life events, a long period of homelessness (1–2 years), and the stigma associated with homelessness.

There was a range of reported attempts at suicide among homeless youth of 40 to 80% [43] and suicidal behaviour among homeless youth of 23% to 67 per cent [12, 43, 60, 61]. Suicidal attempt-rate findings generally range from 20 to 40% [17, 62].

The prevalence in the current study is much higher than those reported in Tel Aviv, Israel, and Vancouver, Canada (5.8% and 9.3%, respectively) [63, 64]. However, the current study’s estimate is lower than those reported in Toronto, Canada, and New York City (62%, 42%, and 46%) [18, 63, 65–67].

The current study differs from the previous study in several ways, including the measurement tool used to measure suicidal ideation and attempt and the cut-off point used to categorize young adults’ suicidal behaviour. Several factors may have contributed to the differences in prevalence, including how suicidal attempts were assessed, the study setting, the study design, the age group, the study year, and the screening tools used. Different factors may explain the differences in suicide behaviour prevalence. Perhaps the difference might be due to the difference in ages. It could be due to differences in study design, duration of homelessness, using different outcomes such as ideation, attempt, or both, as well as the location of the study.

### Factors associated with suicidal behaviour

The study also found several factors related to suicidal behaviour in homeless young people. The length of homelessness was a factor that was significantly associated with suicidal behaviour.

The odds of having suicidal behaviour among homeless youth and young adults 1 to 2 years before data collection time were 2.244 times (AOR = 2.244, 95% CI: (1.447–3.481) more likely to have suicidal behaviour as compared to homeless people who have lived on the street for less than six months. Our findings are also consistent with other studies demonstrating the association between the duration of homelessness and recent suicide attempts [68]. The reason may be stress, substance abuse, or challenges on the streets they face during their extended period of homelessness.

Previous studies have suggested that homeless youth and young adults face intense stigma [18, 43, 66–68]. In addition, homeless related-stigma was significantly associated with suicidal behaviour in this study. Those homeless young people who experienced homeless-related stigma were 1.629 times (AOR = 1.629, 95% CI: (1.149–1.505) more likely to have suicidal behaviour than their counterparts.

The experience of social stigma also revealed significant relationships with solitude, suicidal ideation, and suicidal attempts [43, 68–71]. Homeless people with or without co-occurring mental disorders may be stigmatized by their situation [72]. Due to misconceptions, ignorance, and fears, homeless people have been stigmatized, leading to immense suffering [73]. Stigma can contribute to stereotypes, prejudices, and discrimination [43, 73]. Consequently, they might suffer verbal or physical abuse, which could make them suicidal.

Experiencing stressful life events in the past six months was significantly associated with suicidal behaviour in those participants with medical/surgical illnesses in the past six months. In the past six months, those with a medical or surgical condition were more than 1.655 times (AOR = 1.655, 95% CI: 1.132–2.418) more likely to have suicidal behaviour than those with not experienced any stressful life events.

There are several reasons why physical or surgical conditions lead to suicide. Research has shown that people suffering from physical illnesses often experience emotional stress and chronic pain related to depression and anxiety [74, 75]. Suicidal behaviour may result because of this [75, 76]. Despite the absence of mental illness, conditions that affect an individual’s physical health may contribute to the development of suicidal behaviour [40].

Nevertheless, there is a disagreement between the current study and previous studies [77, 78], which reported a positive correlation between participant age, family history of homelessness, and history of childhood violence. Possible reasons for the difference could be the different study times, the age of the participants, the definition of the homeless group, or possible methodological differences between the two studies.

### Strengths and limitations of the study

This study has few limitations. First, our study used a cross-sectional design that makes it difficult to determine the causality or temporal relationships between suicidal behaviour and its associated factors. Second, the finding of this study may not be generalizable to regions or locations that are not similar to the study sites selected for this research, such as rural areas. Additionally, some homeless individuals may have suffered from impulsive control disorders, bipolar disorder, or schizophrenia, contributing to their suicidal behaviour. This was not evaluated in this study. Nevertheless, depression was taken into account. On the other hand, Several strengths of this study need to be highlighted. First, our study is the first to explore the prevalence and factors associated with suicidal behaviours among street-dwelling homeless youth in Ethiopia, which can be considered a strength. Second, our sample was representative, as it was conducted in several city and town administrations of southern Ethiopia, representing various socio-cultural contexts, which is also considered a strength.

## Conclusions and recommendations

Suicidal behaviour is a significant public health concern among young homeless street dwellers in southern Ethiopia. Suicidal behaviour has a statistically significant relationship with stressful life events, prolonged homelessness (1–2 years) and the stigma related to homelessness in this group. Consequently, our study suggests that policymakers and programme planners should develop a strategy for preventing, detecting and managing suicidal behaviour among street-dwelling homeless young people, and a vulnerable and understudied population. Moreover, an effective community-based suicide prevention campaign must be designed and implemented for homeless street youth in Ethiopia. The next step to addressing the mental health needs of street-dwelling homeless youth is to improve access to mental health care, particularly for those exhibiting suicidal behaviour. Furthermore, we recommend a prospective study to better understand suicidal behaviour among street-dwelling homeless young people and its risk factors.

## Data Availability

The datasets used and analyzed during the current study are available from the corresponding author upon reasonable request.

## Abbreviations

ASSIST: Alcohol, Smoking, and Substance Involvement Screening Test
C-SSRS: Columbia-Suicide Severity Rating Scale
SBQ-R: Revised Suicide Behaviour Questionnaire
SPSS-20: Statistical Package for the Social Sciences-20
